# Investigating the Human Host—ssRNA Virus Interaction Landscape Using the SMEAGOL Toolbox

**DOI:** 10.3390/v14071436

**Published:** 2022-06-29

**Authors:** Avantika Lal, Mariana Galvao Ferrarini, Andreas J. Gruber

**Affiliations:** 1 Insitro, South San Francisco, CA 94080, USA; avantika0290@gmail.com; 2 Univ Lyon, INSA Lyon, INRAE, BF2I, UMR 203, 69621 Villeurbanne, France; mari.ferrarini@gmail.com; 3 Laboratoire de Biométrie et Biologie Évolutive, UMR 5558, CNRS, Université de Lyon, Université Lyon 1, 69622 Villeurbanne, France; 4 Department of Biology, University of Konstanz, Universitaetsstrasse 10, D-78464 Konstanz, Germany

**Keywords:** RNA virus genome, RNA-binding protein, virus-host interaction, toolbox

## Abstract

Viruses have evolved numerous mechanisms to exploit the molecular machinery of their host cells, including the broad spectrum of host RNA-binding proteins (RBPs). However, the RBP interactomes of most viruses are largely unknown. To shed light on the interaction landscape of RNA viruses with human host cell RBPs, we have analysed 197 single-stranded RNA (ssRNA) viral genome sequences and found that the majority of ssRNA virus genomes are significantly enriched or depleted in motifs for specific human RBPs, suggesting selection pressure on these interactions. To facilitate tailored investigations and the analysis of genomes sequenced in future, we have released our methodology as a fast and user-friendly computational toolbox named SMEAGOL. Our resources will contribute to future studies of specific ssRNA virus—host cell interactions and support the identification of antiviral drug targets.

## 1. Introduction

According to Baltimore’s classification, Group IV and Group V viruses have single-stranded RNA (ssRNA) genomes [1]. Whereas (+)ssRNA Group IV viruses package the positive-sense genome that can be directly translated into protein by the translational machinery of the host cell, the (−)ssRNA Group V viruses contain a negative-sense genome that needs to be transcribed into a positive-sense message before translation. ssRNA viruses interact with many host factors in the infected cells in order to facilitate viral replication, subgenomic RNA transcription, and translation of viral proteins. At the same time, host cellular factors detect viral RNA and activate intracellular signalling pathways leading to antiviral responses. Interactions between viral RNAs and host RNA-binding proteins (RBPs) are key to these processes.

ssRNA viruses such as the Hepatitis C virus, the Ebola virus, the Influenza virus, and the SARS-CoV-2 virus responsible for the ongoing COVID-19 pandemic are of high epidemiologic relevance. Understanding how these viruses interact with and impact host cells is key for designing means to combat these infections. A currently prominent example is the SARS-CoV-2 genome, which is bound by hundreds of human proteins [2,3]. More broadly, coronaviruses are known to co-opt human RBPs to promote their stability, translation and replication [4]. Furthermore, viral RNAs may also sequester RBPs to influence gene expression in the host. For instance, the Sindbis virus was found to “sponge” ELAV Like RNA Binding Protein 1 (ELAVL1) molecules via uridine (U)-rich elements in its 3′ untranslated region (UTR) causing changes in splicing, polyadenylation and stability of host messenger RNAs (mRNAs) [5]. Although studies on RBPs and viral genomes point to the importance of RBP interaction networks in viral infections, genome-scale experimental and functional studies are relatively sparse and are cell type and condition specific.

In order to support experimental research in this area with computational predictions, we developed SMEAGOL (Sequence Motif Enrichment And Genome annOtation Library), a Python library to analyse RBP binding motifs in nucleic acid sequences. SMEAGOL identifies proteins whose binding motifs are significantly enriched or depleted in a sequence, thus highlighting the interactions that are most likely under evolutionary selection and functionally significant. By applying SMEAGOL to 197 Group IV and Group V viral genomes we have constructed a comprehensive resource for studying ssRNA virus/RBP interactions.

## 2. Materials and Methods

### 2.1. Curation of Viral Genomes

The complete genome sequences for viruses (Taxonomy ID: 10239) deposited in the NCBI repository (https://www.ncbi.nlm.nih.gov/genome/browse#!/viruses/) (accessed on 6 November 2020) were retrieved using the following search/filter strategies: only RefSeq entries of specific families of (+)ssRNA and (−)ssRNA viruses known to infect humans (host = “human”) were selected. We manually curated these data by adding missing information from additional viral databases ViPR (https://www.viprbrc.org) (accessed on 6 November 2020) [6] and ViralZone (https://viralzone.expasy.org/) (accessed on 6 November 2020) [7]. All information regarding reference strains or the selection of representative strains/genotypes along with excluded genomes can be found in Supplementary Data S1.

The complete genomic sequences were downloaded along with the GFF annotations. For (+)ssRNA viruses, the GFF annotation was used to extract the 3′ UTR and 5′ UTR sequences wherever possible.

### 2.2. Curation of Position Weight Matrices (PWMs)

All available position matrices were downloaded from the ATtRACT database (https://attract.cnic.es/) (accessed on 2 February 2021) and from the RBPDB database (http://rbpdb.ccbr.utoronto.ca/, version 1.3.1) accessed on 2 February 2021. The matrices were filtered to retain only matrices derived from competitive binding experiments using wild-type human RBPs. RBPDB matrices which were redundant with ATtRACT were removed. PWMs from ENCODE RNA Bind-n-Seq assays [8] were constructed using the ENCODE computational pipeline [8] and added to this list.

For RBPDB, position frequency matrices (PFMs) were converted to Position Probability Matrices (PPMs) using the ‘smeagol.matrices.pfm_to_ppm’ function with a pseudocount of 0.01. For ATtRACT, position probability matrices (PPMs) were downloaded directly.

Forty four PPMs were trimmed using the ‘smeagol.matrices.trim_ppm’ SMEAGOL function to remove low-information content positions at the ends of the PPM. This function calculates the information content (IC) for each position in the PPM using the formula:

$$IC=2+\Sigma_{i\epsilon A,C,G,T^{}}p_{i}\;log_{2}\left(p_{i}\right)$$

where *p_i_* is the probability for base *i* at that position. The mean IC across all positions was calculated and positions at the ends of the PPM that had an IC lower than 10% of the mean were dropped. The entropy of each processed PPM was calculated using the ‘smeagol.matrices.entropy’ function. This applies the following formula to each position of the PWM:

$$H=-\Sigma_{i\epsilon A,C,G,T^{}}p_{i}\;log_{2}\left(p_{i}\right)$$

where *i* represents the base. The entropies of all positions in the PWM were then summed.

Outliers with a total entropy greater than 10 were dropped, as were PPMs of length less than four or greater than 12 bases. The processed PPMs were then converted to PWMs using the ‘smeagol.matrices.ppm_to_pwm’ SMEAGOL function. Ultimately, 362 PWMs representing 146 human RBPs were considered for the downstream analyses.

PWMs with a high sequence bias toward one of the four bases (A, G, C, or T/U) were identified by scanning sequences of poly-A, poly-C, etc. PWMs that had a match score of >0.8 to a homopolymer of a specific base were annotated as being biased toward that base.

### 2.3. Selection of Representative PWMs

Eighty four RBPs had multiple PWMs in our filtered final set of PWMs. For 66 of these, we used the ‘smeagol.matrices.choose_representative_pm’ function in SMEAGOL to select a single representative PWM. This function calculates pairwise similarities between all PWMs in the group based on the normalized Pearson correlation metric [9] and selects the one which has the maximum similarity (defined as median normalized correlation) across all the others as a representative PWM.

For 18 RBPs, we observed PWMs falling into dissimilar groups (specifically, the normalized correlation between at least one pair of PWMs for the RBP was below 0.2). Therefore, for each of these 18 RBPs, we used the ‘smeagol.matrices.cluster_pms’ function of SMEAGOL to cluster the PWMs. This function applies agglomerative clustering with complete linkage to the PWMs based on the normalized correlation metric and selects the PWM with the highest median normalized correlation to all others as the representative PWM for each cluster. Agglomerative clustering was first tried for two clusters, and the number of clusters was increased until the minimum pairwise normalized correlation of PWMs within each cluster was at least 0.2. A similar procedure is used by the matrix-clustering tool [9].

### 2.4. Calculation of Motif Enrichment and Depletion

The ‘smeagol.scan.scan_sequence’ function of SMEAGOL scans a given nucleic acid sequence by calculating the PWM match score for each position in the sequence. Specifically, at each position in the sequence, the subsequence of length *k* (where *k* is the length of the PWM) starting at the given position is taken, and the PWM match score is obtained by summing over the PWM log-likelihood ratios at each of the *k* positions, each time selecting the PWM element that corresponds to the nucleotide in the sequence. The score is then divided by the maximum possible score that could be obtained using that PWM [10]. We used this function to scan the downloaded ssRNA virus genome sequences, as well as their reverse complement sequences, with the 362 selected RBP PWMs, and identified putative binding sites with a score threshold of 0.8.

We used the ‘smeagol.enrich.enrich_in_genome’ function in SMEAGOL to calculate a *p*-value for enrichment or depletion of each PWM and viral genome. The *p*-value is calculated as follows. For each genome and PWM combination, SMEAGOL counts the number of predicted binding sites. It then generates 1000 background sequences that have the same nucleotide and dinucleotide frequency as the genome, scans each background sequence, and counts the number of predicted binding sites in the background sequences to generate a background distribution. This is used to calculate the expected probability of finding a binding site in the query sequence based on its sequence composition alone. A two-sided binomial test is used to calculate the *p*-value, which is then adjusted for multiple-testing using the Benjamini–Hochberg correction. For multi-segmented viral genomes, SMEAGOL calculates a single enrichment score across all segments.

PWMs with FDR-adjusted *p*-values < 0.05 were considered to be significantly enriched/depleted. The ratio (fold change) of the real and expected number of binding sites in the query sequence was used as a measure of effect size.

### 2.5. Calculation of Motif Enrichment and Depletion in Genomic Windows

Local window enrichment plots were generated using the ‘smeagol.enrich.enrich_in_sliding_windows’ function of SMEAGOL. This function creates windows tiling over the entire genome (for the figures here, non-overlapping windows of 500 bp were used) and tests whether the number of predicted binding sites for an RBP in each window is significantly higher/lower than the expected number based on a model in which binding sites for the RBP are uniformly distributed across the genome. *p*-values were calculated using a two-sided Fisher’s exact test and adjusted using the Benjamini–Hochberg procedure.

### 2.6. Variant Effect Prediction

We downloaded information on 36,688 SARS-CoV-2 mutations from the GESS database (https://wan-bioinfo.shinyapps.io/GESS/) accessed on 14 September 2021. We used the ‘smeagol.variant.variant_effect_on_sites’ function in SMEAGOL to predict the impact of each of these variants on the PWM match score in the surrounding region, for PWMs representing ten selected RBPs. This function selects mutations that intersect with the predicted binding sites of the selected RBPs and calculates the PWM match score of each predicted binding site using first the reference sequence and then the alternate sequence. We selected variants that reduce the PWM match score of a binding site to less than 0.5 as potential site-disrupting variants.

## 3. Results

### 3.1. Identification of Sequence Motif Enrichment/Depletion Using SMEAGOL

**Figure 1:**
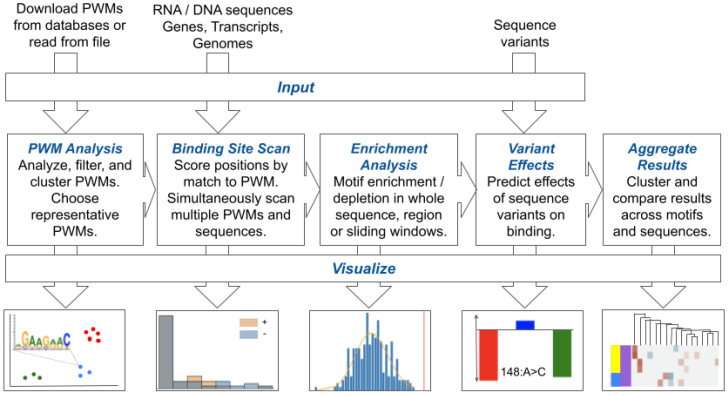
SMEAGOL enables the investigation of regulatory binding motif enrichment and variant effects in viral genomes. SMEAGOL takes as input sequence files in FASTA format and regulator binding specificities in the form of position weight matrices (PWMs) in order to perform PWM analysis, sequence scanning, enrichment/depletion analysis, and variant effect prediction. Finally, SMEAGOL enables visualization of the results in various ways.

SMEAGOL (https://github.com/gruber-sciencelab/SMEAGOL) is a Python library designed for comprehensive motif occurrence analysis in nucleic acid sequences using PWMs, which can represent the binding specificity of a variety of nucleic acid-interacting regulators. SMEAGOL can directly load PWMs that represent RBP binding specificities from the ATtRACT and RBPDB databases [11,12]. As curated databases of RBP binding motifs typically contain PWMs of different quality, SMEAGOL also includes modules to analyse, filter, compare, cluster, and visualize PWMs. Moreover, SMEAGOL enables scanning of sequences with the curated PWMs and filtering of these results. Post-processing modules enable the calculation and visualization of statistical enrichment or depletion of sequence motifs, as well as predicted effects of sequence variants on PWM sites. An overview of the SMEAGOL functionalities is provided in Figure 1.

### 3.2. The Genomes of ssRNA Viruses Show Evidence of Selection of RBP Binding Sites

To find out which RBPs are enriched/depleted in binding sites across ssRNA virus genomes, we have used PWMs representing the experimentally determined sequence binding preferences of human RBPs from the ATtRACT [12], RBPDB [11], and ENCODE [8] databases. We have used SMEAGOL to filter and curate these motifs (Methods, Supplementary Figure S1) and obtained a curated set of 362 PWMs representing the binding specificities of 146 human RBPs. We then used this set of PWMs and SMEAGOL to scan the complete genome sequences of 197 ssRNA viruses belonging to 19 families (Table 1, Supplementary Data S1) to identify putative RBP binding sites (Supplementary Data S2).

To find evidence of evolutionary selection for or against RBP binding to viral RNA, we determined the enrichment or depletion of RBP binding motifs in each viral genome compared to dinucleotide-randomized versions of the genome (Supplementary Data S3). It should be noted that, although the viruses in our study have single-stranded genomes, the complementary strand of the genome is also synthesized during the viral life cycle. We therefore repeated this procedure for the complementary strand of each genome.

**Figure 2:**
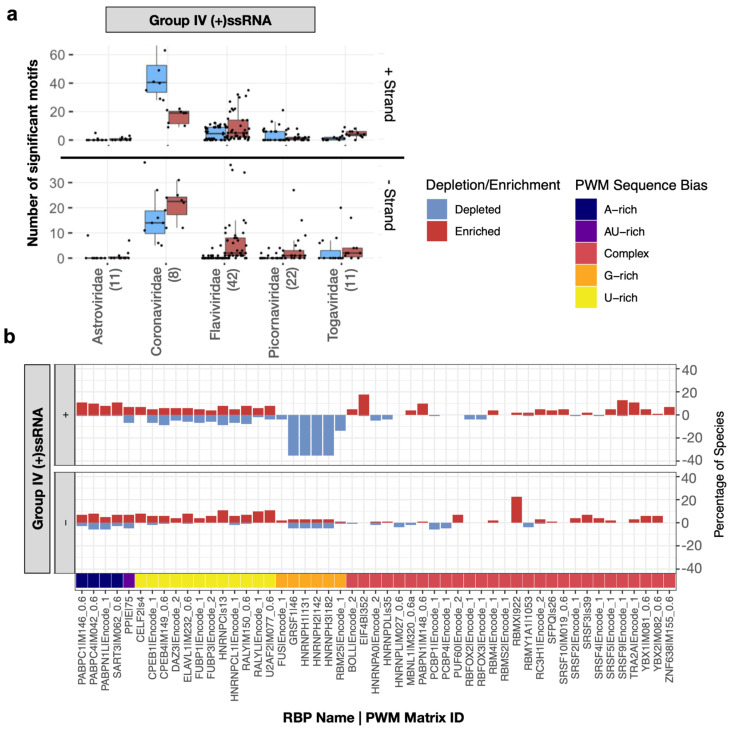
SMEAGOL uncovers RBPs whose binding motifs are enriched or depleted in ssRNA virus genomes. (**a**) Number of motifs significantly enriched or depleted in each family of Group IV viruses. The number of genomes per family is given in parentheses. Families with more than five genomes in our dataset were included. Box plots are defined as follows: centre line, median; box limits, upper and lower quartiles; whiskers, 1.5× interquartile range. Individual data points are also shown. (**b**) Percentage of viral genomes with significant (two-sided binomial test, FDR-adjusted *p*-values < 0.05) enrichment (in red) and depletion (in blue) per PWM, separated by viral genome strand. For readability, shown are only representative PWMs that had more than three significant enrichment/depletion events. The PWM sequence bias (see Methods) is presented on the bottom of the plot. While some PWMs have a more complex sequence (light red), others are rich in single nucleotides (A-rich in navy blue, AU-rich in purple, U-rich in yellow, and G-rich in orange). The results for Group V viruses can be found in Supplementary Figure S5 and a comprehensive figure that contains all representative PWMs is provided as Supplementary Figure S6.

Within the (+)ssRNA (Group IV) virus genomes, we found that the number of motifs enriched or depleted varied significantly between families (Figure 2a). While some RBP binding motifs are generally depleted or enriched in (+)ssRNA virus genomes, other motifs are much more specific to a subset of viruses. For instance, G-rich motifs recognized by several splicing factors (GRSF1, HNRNPH1, HNRNPH2, HNRNPH3, HNRNPF, HNRNPA2B1) are frequently depleted in the plus-strand of (+)ssRNA virus genomes across multiple families (Figure 2b and Supplementary Data S3).

**Figure 3:**
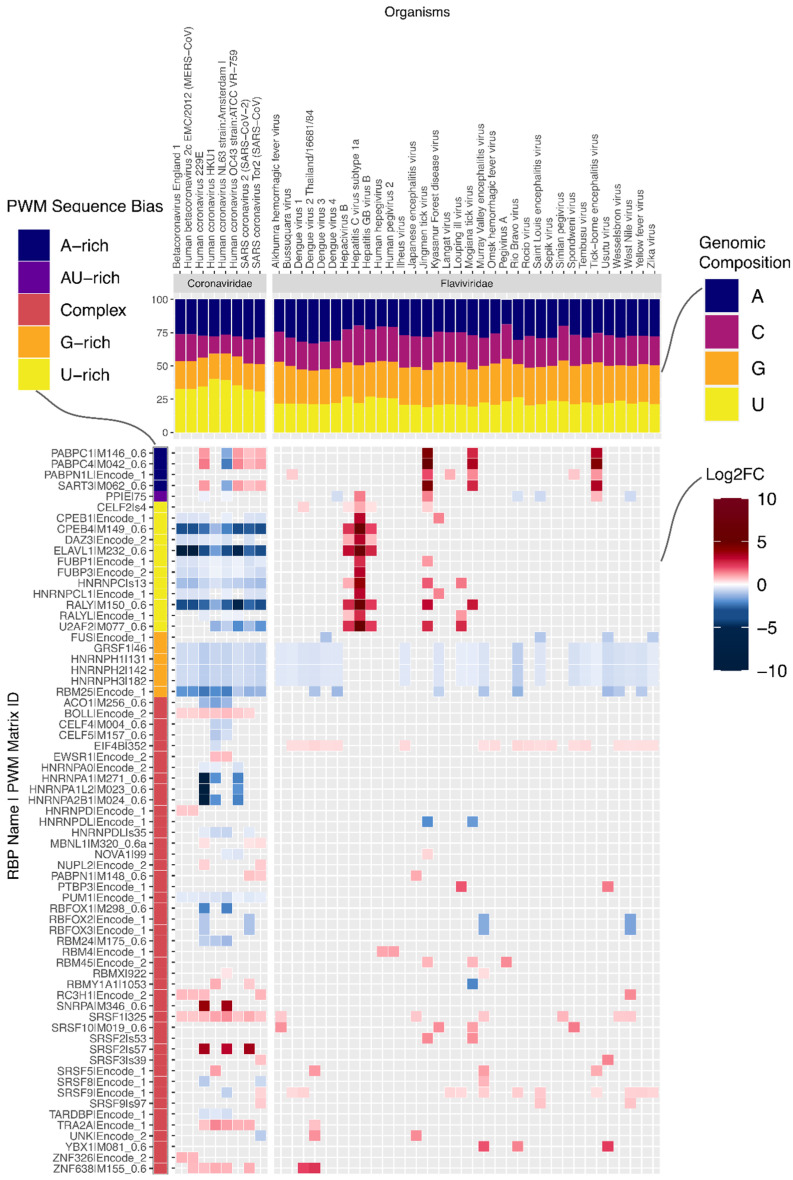
Enrichment and depletion results for Coronaviridae and Flaviviridae families. Heatmap with results for positive sense molecules in terms of log2 fold change (Log2FC) of enrichment (in red hues) or depletion (in blue hues) for single genomes within two (+)ssRNA families. The PWM sequence bias is presented on the left side of the plot (as explained in Figure 2). The nucleotide compositions of the viral genomes are provided on the top of the heatmap (A in navy-blue, C in magenta, G in orange, U in yellow). For reasons of space, representative PWMs enriched or depleted in more than one viral genome are shown (species-specific results are provided in Supplementary Figure S7). For a full list of motifs enriched and depleted in all viral genomes, see Supplementary Data S3.

On the other hand, the motif for RBMX is enriched on the negative sense molecule in 24 genomes belonging to three Group IV families. For example, all four Dengue virus (DENV) genomes in our dataset showed enrichment in the negative sense molecule for RBMX (Supplementary Data S2). Consistently, RBMX was reported to be required for efficient amplification of DENV and its knockdown significantly decreases the titre of DENV [14]. The second most commonly enriched motif was related to the translation factor EIF4B, specifically in the positive strand of (+)ssRNA viruses. EIF4B was reported to bind to DENV RNA [15] and its depletion reduced the efficiency of translation initiation in Zika virus (ZIKV) [16]. All DENV and ZIKV genomes in our dataset had significant enrichment for this host factor as did most flaviviruses (Figure 3).

Having observed significant differences between viral families, we next examined prominent families individually. Coronaviruses, which have the longest genomes of all viruses in our dataset (Supplementary Figure S2, one-sided Wilcoxon rank-sum test U statistic = 1536, *p* = 8.5 × 10^−7^), also show more enrichment and depletion of binding motifs than any other family (Figure 2a). Strikingly, the number of RBP motifs depleted on the plus strand is much higher than the number of enriched motifs. It is conceivable that given their long genomes, coronaviruses must actively prevent being bound by non-beneficial host RBPs. On examination, we found that the striking number of depleted motifs in these genomes reflects depletion of U-rich elements (UREs) bound by RBPs such as HNRNPC, RALY, CELF2, TIA1, ELAVL1 and PPIE (Figure 3). This is despite coronaviruses being the most U-rich of all viruses in our dataset (Figure 3, Supplementary Figure S3, one-sided Wilcoxon rank-sum test U statistic = 1530, *p* = 5.4 × 10^−13^). By contrast, a subset of flaviviruses, which are relatively poor in uridines, are enriched for these UREs (Figure 3, Supplementary Figure S3). In addition to the depletion of UREs and G-rich elements, all eight coronaviruses in our dataset showed enrichment of motifs for the SRSF1 splicing factor and slight depletion for a PUM1-associated motif. Interestingly, the PUM1 RBP was reported to reduce mRNA stability [17].

Regarding the Group V viruses, it is important to note that these viral genomes do not exist as naked RNA, as they encode nucleoproteins which encapsidate the entirety of the (+) and (−) strand molecules to form stable ribonucleoprotein complexes [18]. Nevertheless, the mRNAs transcribed from their genomes, including UTR regions, are not complexed with nucleoproteins, and thus might be available for host RBP interaction. Consistent with this, we found that although we detect enrichment and depletion of some motifs in (−)ssRNA (Group V) viral genome sequences, the number of both enriched and depleted motifs was much lower for both strands compared to (+)ssRNA (Group IV) viral genomes (Supplementary Figures S4–S6).

### 3.3. Non-Coding Regions in Viral Genomes Show Distinct Patterns of Enrichment

Like cellular mRNAs, the genomes of (+)ssRNA viruses also contain 5′ and 3′ UTRs, which have been shown to bind host RBPs. While the 5′ UTR contains elements that regulate the efficiency and timing of translation initiation and viral replication, host factors binding to the 3′ UTR can be critical to many aspects of the life cycle of a virus, including but not limited to RNA replication and stability. Host RBPs also mediate 5′ UTR—3′ UTR interactions, resulting in ‘circularization’ of the viral genome [19,20].

As the UTR regions have distinct regulatory functions from the remaining genome and their sequences are not constrained to code for proteins, we reasoned that they may be enriched for binding sites of specific RBPs relevant to their functions. These enrichments may not be detectable over the whole genome, and indeed may be cancelled out since it may be detrimental for some UTR-specific proteins to bind elsewhere in the genome. We therefore repeated the analysis specifically for 5′ and 3′ UTR sequences of 89 (+)ssRNA viruses whose UTR positions were annotated.

**Figure 4:**
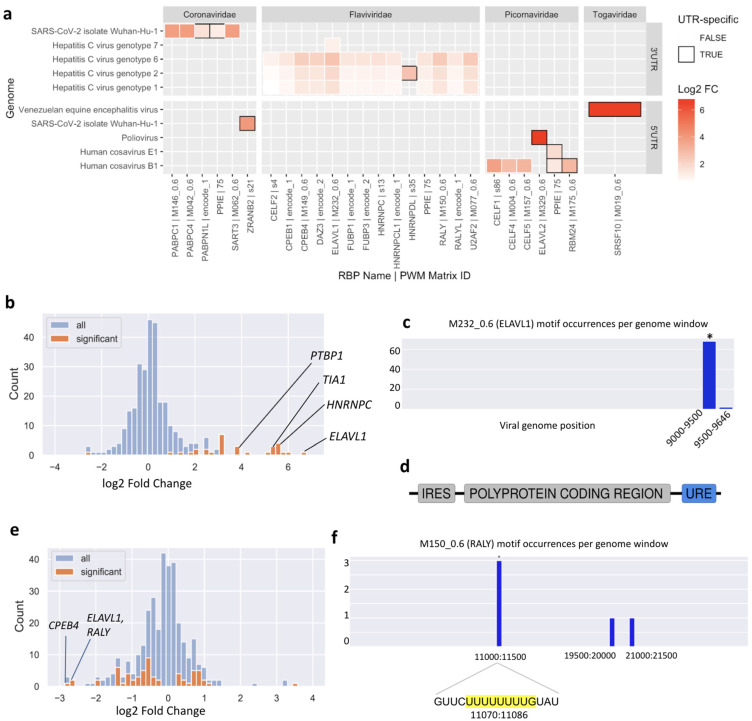
Motif enrichment patterns in specific regions of viral genomes. (**a**) UTR enrichment/depletion analysis results from SMEAGOL. Cells with black frames represent motifs that were not significantly enriched in the whole genome. Only representative motifs (see Methods) were included in this figure. Full results are given in Supplementary Data S4. (**b**) Motifs for 22 RBPs were predicted by SMEAGOL to be significantly enriched within the HCV genome while one motif was depleted (shown are the results for HCV genotype 1a). (**c**) Using the local window enrichment function of SMEAGOL (see Methods), we found that the vast majority of regions lack binding motifs for the ELAVL1 RBP, whereas it was highly enriched (marked by an asterisk) within a region at the 3′ end of the HCV genome (Two-sided Fisher’s exact test, odds = 21.8, FDR-adjusted *p*-value = 1.4 × 10–51). (**d**) A U-rich element (URE) is located within the 3′ UTR of the HCV genome [32]. (**e**) RBPs predicted by SMEAGOL to be significantly enriched/depleted in binding sites within the SARS-CoV-2 genome. URE-binding RBPs are shown to be most strongly depleted. (**f**) Using the local window enrichment function of SMEAGOL, we observed that binding motifs of RALY are absent in most of the SARS-CoV-2 genome but enriched in a specific region (Two-sided Fisher’s exact test, odds = 36.5, FDR-adjusted *p*-value = 0.013). On closer inspection, this is due to multiple motifs within an URE at position 11074.

This analysis highlighted new putative host-virus associations. Of 116 enriched motif-UTR pairs (Supplementary Data S4), 22 were not enriched in the whole genome of the same virus. In general, both 3′ UTRs and 5′ UTRs are enriched in binding motifs of specific RBPs. One consistent result was an enrichment of U-rich motifs in the 3′ UTRs of multiple Hepatitis C Virus (HCV) genotypes (Figure 4a).

To further validate the predictions made by SMEAGOL, we focused on two well-studied pathogenic (+)ssRNA viruses, namely HCV, from the Flaviviridae family, and SARS-CoV-2, from the Coronaviridae family. The interactions of these viruses with host factors have been studied experimentally and both are enriched/depleted in binding sites of specific RBPs according to our analysis.

### 3.4. The Hepatitis C Virus Genome Is Highly Enriched in Binding Sites of U-Rich Element Binding RBPs

We found motifs for 23 RBPs to be significantly enriched or depleted (FDR-adjusted *p*-value < 0.05) in the HCV genome (Figure 4b) and ranked these by their absolute fold change. Among the top ten significant RBPs, four interactions have already been experimentally validated.

The RBP that is most highly enriched in binding sites in the HCV genome is ELAVL1, also called HuR. Multiple experimental studies have reported direct binding of ELAVL1 to the HCV genome and siRNA experiments have shown that ELAVL1 knockdown counteracts HCV replication [21,22,23,24]. Using the local enrichment function of SMEAGOL we found that the HCV genome is highly enriched in ELAVL1 binding motifs within its 3′ UTR (Figure 4c), consistent with previous reports that ELAVL1 directly interacts with a U-rich region located within the 3′ UTR of the virus [22,24] (Figure 4d). The Polypyrimidine Tract Binding Protein 1 (PTBP1) was also one of the most enriched RBPs predicted by SMEAGOL and there exists experimental evidence for its binding to the 3′ UTR of the virus [25,26]. Another highly enriched RBP that was previously reported to directly interact with HCV was the Heterogeneous Nuclear Ribonucleoprotein C (HNRNPC). HNRNPC binds to the poly(U) tract in the HCV 3′ UTR [27], and an siRNA study has shown that *HNRNPC* knockdown decreases cellular HCV RNA levels suggesting that *HNRNPC* might positively contribute to HCV replication [23]. Finally, the TIA1 Cytotoxic Granule Associated RNA Binding Protein was also among the most enriched RBPs predicted by SMEAGOL. Studies have shown that TIA1 is required for efficient HCV infection [28] and that it interacts with the HCV 3′ UTR [22].

The remaining six RBPs whose motifs are most significantly enriched in the HCV genome are RALY, CPEB4, HNRNPCL1, TRNAU1AP, U2AF2, and BOLL. Because HCV infection is liver-specific, we checked for expression of these proteins in the liver according to the Human Protein Atlas [29], and found that all except BOLL are expressed. As BOLL binds U-rich motifs similar to those of ELAVL1, we suggest that the enrichment for BOLL motifs is incidental. Further down the list of significant RBPs, we see more interesting candidates enriched in the HCV genome (Supplementary Data S3), including FUBP1 (rank 19) and YBX1 (rank 20). FUBP1 was reported to facilitate persistent replication of HCV by regulating p53 [30], and there exists experimental evidence for direct interaction of YBX1 with the HCV genome [22,31].

### 3.5. Motif Enrichment Expands upon Functional Studies in the SARS-CoV-2 Genome

The enrichment and depletion of RBP motifs in the SARS-CoV-2 genome is largely like that of the other coronaviruses in our dataset. However, it is unique in having strong enrichment for binding motifs of YBX1 (motif ‘s54’, Supplementary Data S3). Like other coronaviruses, more RBPs were enriched and depleted (18 and 32 respectively) in the positive sense genome sequence compared to the negative sense intermediates (14 and 13 respectively), suggesting that more functional interactions happen with the positive sense molecule. The observation that overall depletion is more common than enrichment suggests that SARS-CoV-2 has more antiviral interactions with human RBPs than pro-viral interactions. This prediction is consistent with experimental observations from CRISPR screens [2].

To place our predictions for specific RBPs in the context of experimental data, we collected a list of proteins that have been experimentally validated to bind to SARS-CoV-2 RNA in infected human or monkey cell lines in three studies [3,33,34] (Supplementary Data S5). PWMs for 41 of these were included in our study, and we computationally predicted binding sites for 40 of these 41 in the SARS-CoV-2 genome (Supplementary Data S3). We found motifs for eight of these RBPs to be enriched while 13 were depleted, indicating that while some interacting RBPs bind to longer regions or an abundance of locations in the viral genome, others are overall depleted in binding sites perhaps in order to minimize antiviral effects or to guarantee highly specific binding to well defined genomic loci.

We compiled a list of experimentally validated antiviral and pro-viral RBPs from CRISPR or siRNA screens in SARS-CoV-2 infected cells [33,35] (Supplementary Data S6). PWMs for 17 known antiviral and four known pro-viral RBPs were included in our dataset. While we did not observe motif enrichment or depletion for the pro-viral proteins, motifs for four of the 17 antiviral proteins (RALY, ELAVL1, FUBP3, PCBP2) were depleted in the SARS-CoV-2 genome, whereas only two were enriched, suggesting that the viral genome may have evolved to avoid interaction with defensive host proteins, where possible. Motifs for an additional four antiviral RBPs (HNRNPA2B1, DAZAP1, TARDBP, PPIE) were also depleted at a more permissive FDR-adjusted *p*-value threshold of 0.1. Out of these, RALY, ELAVL1, FUBP3 and PPIE bind to UREs. Interestingly, although the predicted binding sites for numerous URE-binding RBPs are strongly depleted overall in the SARS-CoV-2 genome (Figure 4e), the few binding sites that are predicted are significantly concentrated within a region in the NSP6 gene. In particular, an URE at position 11074 contains three of five predicted binding sites for the antiviral RBPs RALY and ELAVL1 (Figure 4f).

Computational studies offer an opportunity to predict novel interactions that may not have been covered in the limited range of cell types and conditions that were studied experimentally. We identified strong enrichment (FDR-adjusted *p*-value < 0.05 and fold change ≥ 2) of motifs for five RBPs (SART3, PABPC1, NUPL2, SRSF2, ZRANB2) and strong depletion (FDR-adjusted *p*-value < 0.05 and fold change ≤ 0.5) of motifs for ten RBPs (CPEB4, HNRNPA1, HNRNPC, HNRNPCL1, HNRNPK, RBFOX1, RBFOX2, RBFOX3, RBM25, U2AF2) that were not listed as hits in the screens we examined.

### 3.6. SMEAGOL Offers Functionality to Predict Sequence Mutation Effects on RBP Binding

Because computational analysis allows us to predict the probable locations of protein binding on the viral genome, it also offers the possibility of predicting how mutations may affect these binding sites. To demonstrate this functionality, we selected ten RBPs that (1) have PWMs in our dataset, (2) are experimentally determined to be antiviral in SARS-CoV-2 infection and/or are shown by our analysis to be significantly depleted in the SARS-CoV-2 genome, and (3) have fewer than ten predicted binding sites on the SARS-CoV-2 genome. These are CPEB4, RALY, ELAVL1, HNRNPA1, RBFOX1, HNRNPK, HNRNPA2B1, DAZAP1, SRSF7 and PCBP2. We hypothesized that mutations that disrupt the binding sites of these RBPs may enable SARS-CoV-2 to escape host antiviral defences. Using SMEAGOL to analyse a database of SARS-CoV-2 mutations [36], we identified 170 mutations that are predicted to disrupt motifs for the selected RBPs (Supplementary Data S7). Interestingly, this list includes 22 non-exonic and 60 synonymous mutations.

As an example, the T11078C (nsp6:p.F36L) mutation, one of the lineage-determining mutations in the N.9 Variant of Interest found in Brazil [37], is predicted to disrupt binding of RALY, ELAVL1, and CPEB4 (Supplementary Figure S8) to the URE at position 11074 (Figure 4f). As discussed above, this URE is one of very few regions predicted to bind to the known antiviral RBPs RALY and ELAVL1, as well as CPEB4 which is validated to bind to the SARS-CoV-2 genome. Interestingly, the much less common T > G mutation at the same position is predicted to have a lesser effect on RBP binding (Supplementary Figure S8). This example illustrates the capability of SMEAGOL to generate predictions for the functional effects of sequence mutations or variants, and to prioritize variants for experimental studies.

## 4. Discussion

There exist several web servers [38] and libraries [10,39,40,41] to scan nucleic acid sequences with PWMs and identify putative binding sites. Several tools [38,41] also calculate a *p*-value for motif enrichment that takes into account the nucleotide composition of the sequence. With SMEAGOL, we aim to provide a unified python-based framework for visualization, analysis, and clustering of PWMs, binding site discovery, variant effect prediction on RBP binding, and binding site enrichment/depletion calculations using a background model that incorporates k-mer shuffling as specified by the user. For instance, dinucleotide shuffling is considered to be more conservative compared to mononucleotide shuffling, as it better accounts for RNA structural features and genomic biases in the occurrence of dinucleotides.

To date, RBP-virus interactions and their relevance have been experimentally measured for only a fraction of RBPs and RNA viruses. SMEAGOL aims to provide computational predictions that can assist biologists in prioritizing promising candidates for experimental testing out of the large number of RBPs present in humans [42]. Computational predictions may be particularly useful for viruses and RBPs with limited experimental data, and further enables us to identify trends and commonalities across all sequenced RNA virus genomes, including many for which experimental data is largely unavailable.

SMEAGOL is designed to efficiently scan large numbers of sequences in parallel as fast or faster compared to existing tools (Supplementary Figure S9), which allows it to perform statistical testing for enrichment or depletion with hundreds of PWMs on large (10 kb) sequences within minutes (Supplementary Figure S10). While SMEAGOL was designed with a focus on RBP-RNA interactions, it can be applied to genomes, genomic regions, genes, or transcripts. Here, we have applied it to perform the first large-scale computational analysis of interactions between RNA viruses and human RBPs.

We found numerous RBP-binding motifs to be enriched or depleted in ssRNA viruses, including motifs that were enriched or depleted globally as well as in a family- or species-specific manner. The RBPs bound by these motifs include host splicing factors as well as RBPs that are known to regulate RNA stability. We report differences in predicted host interactions between viral families, with coronaviruses showing the highest levels of motif enrichment and depletion in their genomes. Coronaviruses may have evolved to avoid being bound by specific RBPs as, given their length, most RBPs will bind the viral genome by chance in the absence of active selection against it. Further, we find an interesting pattern in the occurrence of UREs which bind numerous RBPs that regulate viral infection. These UREs are depleted in the genomes of coronaviruses (which are highly U-rich overall) and enriched in a few flaviviruses including HCV (which are overall depleted in uridine). Consistent with this, we find numerous URE-binding RBPs to be enriched for motifs in the HCV genome, specifically in U-rich elements in the 3′ UTR of the HCV genome [32]. Four of these (HNRNPC [27], PTBP1 [25,26], ELAVL1 [22,24], and TIA1 [22]) have been experimentally shown to bind to the HCV 3′ UTR sequence. Further, it was reported that TIA1 is required for efficient HCV infection [28], and silencing of HNRNPC, PTBP1, or ELAVL1 has been shown to impair HCV replication [21,22,23,24,27], indicating that a multitude of URE-binding RBPs, including ELAVL1, have pro-viral effects on HCV.

In contrast, CRISPR knockout of ELAVL1 sensitized VeroE6 cells to SARS-CoV-2 infection [35], indicating an antiviral effect of the RBP against SARS-CoV-2, though the mechanism is unclear. We previously published an analysis of motif enrichment in the SARS-CoV-2 genome using a similar procedure [43]. Here, we improve upon the previous findings with a more rigorous statistical procedure including dinucleotide shuffling, using an expanded dataset of PWMs, and by placing the results in the context of other Coronavirus genomes and more recent functional studies. Our analysis supports the observation that SARS-CoV-2 is more likely to form antiviral interactions with RBPs than pro-viral ones. Further, we find depletion of motifs for several known antiviral RBPs on the SARS-CoV-2 genome. We extend functional studies by providing binding site predictions for known pro-viral and antiviral RBPs as well as predicting putative interactions. For example, it is interesting that SARS-CoV-2, unlike other coronaviruses, has strong enrichment for binding motifs of YBX1, which has been experimentally validated to bind to SARS-CoV-2 RNA [2] and supports infection by other viruses, including Dengue Virus [44], Influenza [45], and HIV [46]. Finally, we predict which mutations may disrupt the binding sites of known antiviral proteins. While mutations affecting SARS-CoV-2 protein sequences have been extensively studied, the effects of other classes of mutations are less clear. SMEAGOL supports the creation of testable hypotheses on RBP-virus interactions and helps to prioritize non-coding and synonymous mutations for further investigations.

Our dataset of predicted RBP-virus interactions is available (Supplementary Datas S3 and S4) along with our software. We suggest that the proteins highlighted in our analysis can be prioritized in knockout, knockdown, or overexpression studies to experimentally measure their impact on viral pathogenesis. We hypothesize that RBPs whose binding motifs are enriched in viral genomes are more likely to be host factors co-opted by the virus and therefore targets for antiviral drugs, while those showing depletion are more likely to participate in antiviral responses or might have highly position specific functionality. RBPs greatly contribute to host cell RNA regulation. They are well known to mediate mRNA stability [47] and frequently co-regulate mRNA splicing and polyadenylation [48]. Thus RBP-virus interactions may indirectly modulate the transcriptional program of the host cell by sequestering RBPs from host RNAs. For instance, a study has reported that the sequestration of the ELAVL1 RBP by a (+)ssRNA virus causes changes in host cell mRNA stability, splicing and polyadenylation [5]. Future studies may characterize such effects by investigating whether host cell transcripts regulated by virus interacting RBPs exhibit differential expression or processing upon viral infection. Such follow-up studies may benefit from examining the tissue-specific expression of highlighted proteins, in cases where the investigated virus is well known to infect only a limited number of cell types or tissues, as is the case for HCV.

Differences in the vulnerability of cells and tissues in the human body, as well as differences in the susceptibility of individuals to viral disease, may be partially explained by the expression levels of host factors that are interacting with the virus [49]. Thus, in future our work predicting host factors that are relevant to diverse viruses may also contribute to a better understanding of within-individual and between-individual diversity in infection response.

PWM sets of increased quantity and quality will further improve the predictions in the future. An additional approach that may help to further refine our computational predictions would be to consider the viral RNA structure and integrate information on the structural binding requirements of specific RBPs. Finally, deep learning methods have shown promise for identifying nucleic acid-protein binding sites, potentially with higher accuracy than PWM scanning [50,51,52], and tools have recently been developed to learn motif representations from these models [53]. However, trained models are not available for many human RBPs, and the methods are generally difficult to use for non-experts. A possible extension of SMEAGOL in the future could be to incorporate deep learning and RNA structure-based binding models to offer improved predictions wherever possible.

## Figures and Tables

**Table 1:** Viral classes and families as well as the number of species and genomes considered within this study.

Baltimore Classification	Type	Genome Info	Family	Number of Genomes	Number of Species
**Group IV**	(+) ssRNA	Segmented	Flaviviridae *^1^	2	2
		Monopartite	Astroviridae	11	11
			Caliciviridae	2	2
			Coronaviridae	8	8
			Flaviviridae *^1^	37	31
			Hepeviridae	1	1
			Matonaviridae	1	1
			Picornaviridae	22	21
			Togaviridae	11	11
		**Total**		**95**	**88**
**Group V**	(−) ssRNA	Segmented	Arenaviridae	9	9
			Hantaviridae	9	9
			Nairoviridae	3	3
			Orthomyxoviridae	10	4
			Peribunyaviridae	22	22
			Phenuiviridae	10	10
		Monopartite	Bornaviridae	3	2
			Filoviridae	7	6
			Paramyxoviridae	15	15
			Pneumoviridae	2	2
			Rhabdoviridae	12	12
		**Total**		**102**	**94**

*^1^: These two segmented genomes belong to the jingmenviruses tentatively classified in the Flaviviridae family [13].

## Data Availability

All results generated in this study are available in the supplementary materials. Accession numbers for external datasets used in this study are given in Supplementary Data S1. Our curated lists of PWMs (both full and representative sets) and our scripts are available online https://github.com/gruber-sciencelab/VirusHostInteractionAtlas (accessed on 18 June 2022). Figure 2a,b, Figure 3 and Figure 4b,e are based on raw data in Supplementary Data S3. Figure 4a is based on raw data in Supplementary Data S4. Figure 4c,f are based on raw data in Supplementary Data S2. SMEAGOL is available online https://github.com/gruber-sciencelab/SMEAGOL (accessed on 18 June 2022) under an MIT open-source license. Its documentation is available online https://gruber-sciencelab.github.io/SMEAGOL/ (accessed on 18 June 2022) and an introductory tutorial is available online https://github.com/gruber-sciencelab/SMEAGOL/blob/master/vignette_1.ipynb (accessed on 18 June 2022).

## Supplementary Materials

The following supporting information can be downloaded at https://www.mdpi.com/article/10.3390/v14071436/s1. Supplementary Figures S1–S10, Supplementary Data S1–S7.
