# Supplementary material for: Investigating the Human Host—ssRNA Virus Interaction Landscape Using the SMEAGOL Toolbox
Source: Viruses. 2022 Jun 29;14(7):1436. doi: 10.3390/v14071436 (PMC9317827; doi:10.3390/v14071436)
Supplement: Supplementary file 1 [file viruses-14-01436-s001.zip › Supplementary_Materials.pdf]

Supplementary Figures

Avantika Lal [1], Mariana Galvao Ferrarini [2,3], Andreas J. Gruber [4,*]

[1] Insitro, South San Francisco, CA 94080, USA
[2] Univ Lyon, INSA Lyon, INRAE, BF2I, UMR 203, 69621 Villeurbanne, France
[3] Laboratoire de Biométrie et Biologie Évolutive, UMR 5558, CNRS, Université de Lyon, Université Lyon 1, 69622, Villeurbanne, France
[4] Department of Biology, University of Konstanz, Universitaetsstrasse 10, D-78464 Konstanz, Germany

* Correspondence: gruber-at-uni-konstanz.de.

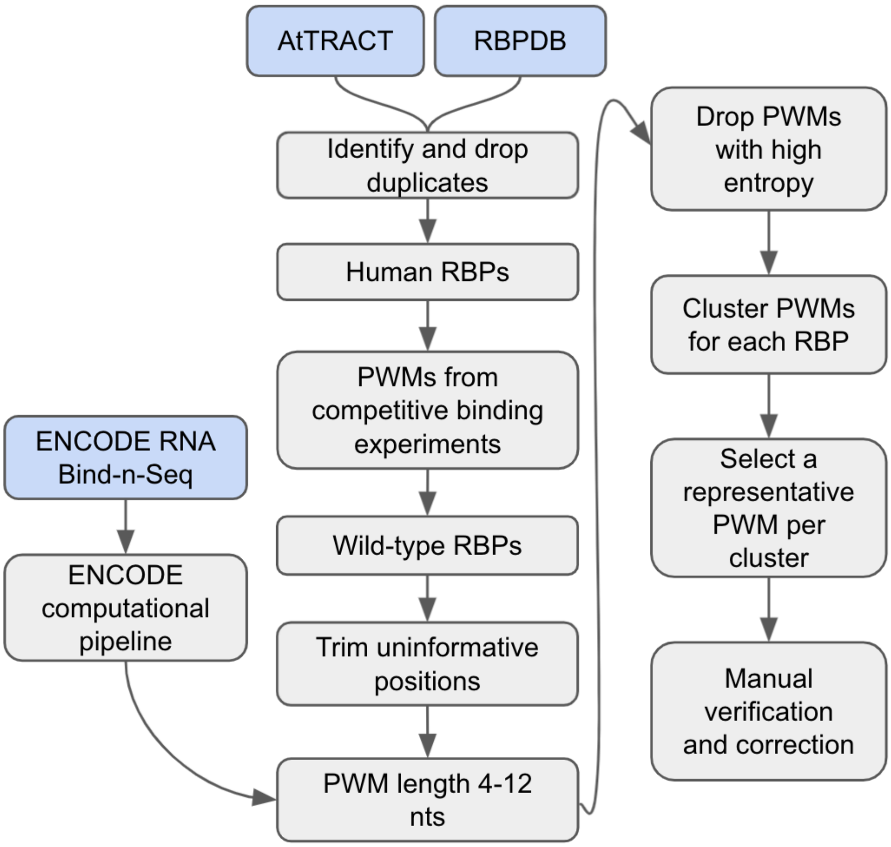

Supplementary Figure S1: Schematic of PWM curation strategy.

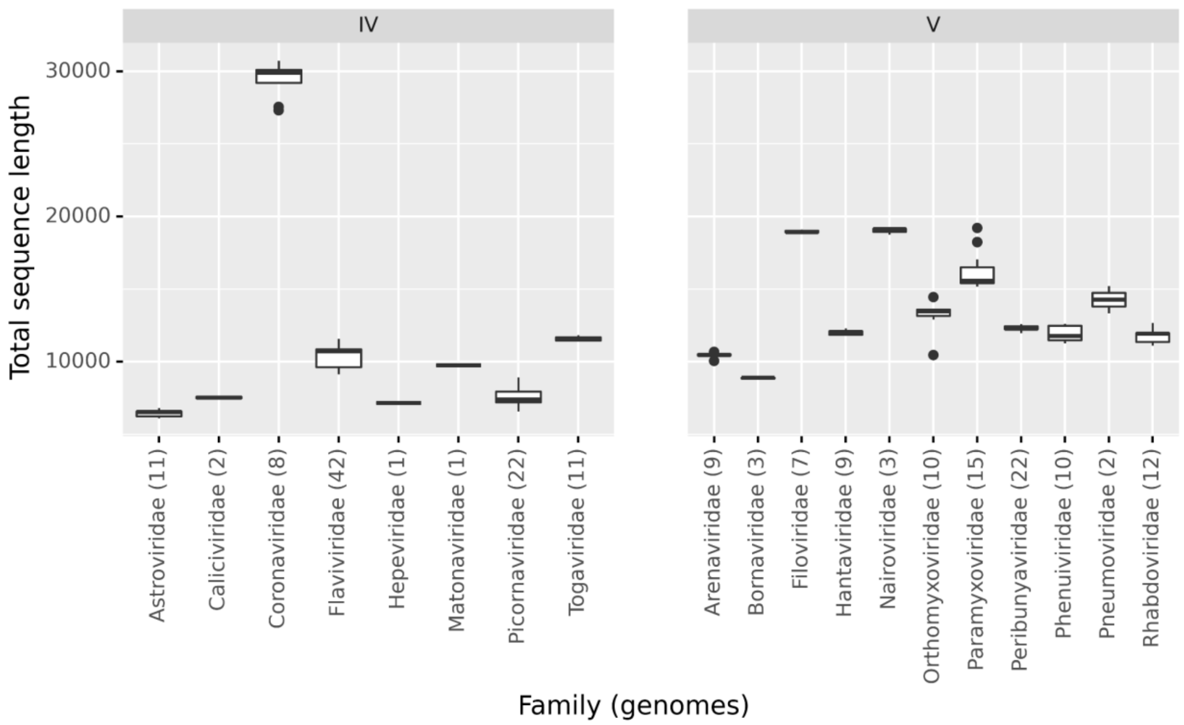

Supplementary Figure S2: Boxplots showing genome lengths in different viral families. The number of genomes for each family is given in parentheses. Box plots are defined as follows: center line, median; box limits, upper and lower quartiles; whiskers, 1.5x interquartile range; points, outliers.

Genomic composition

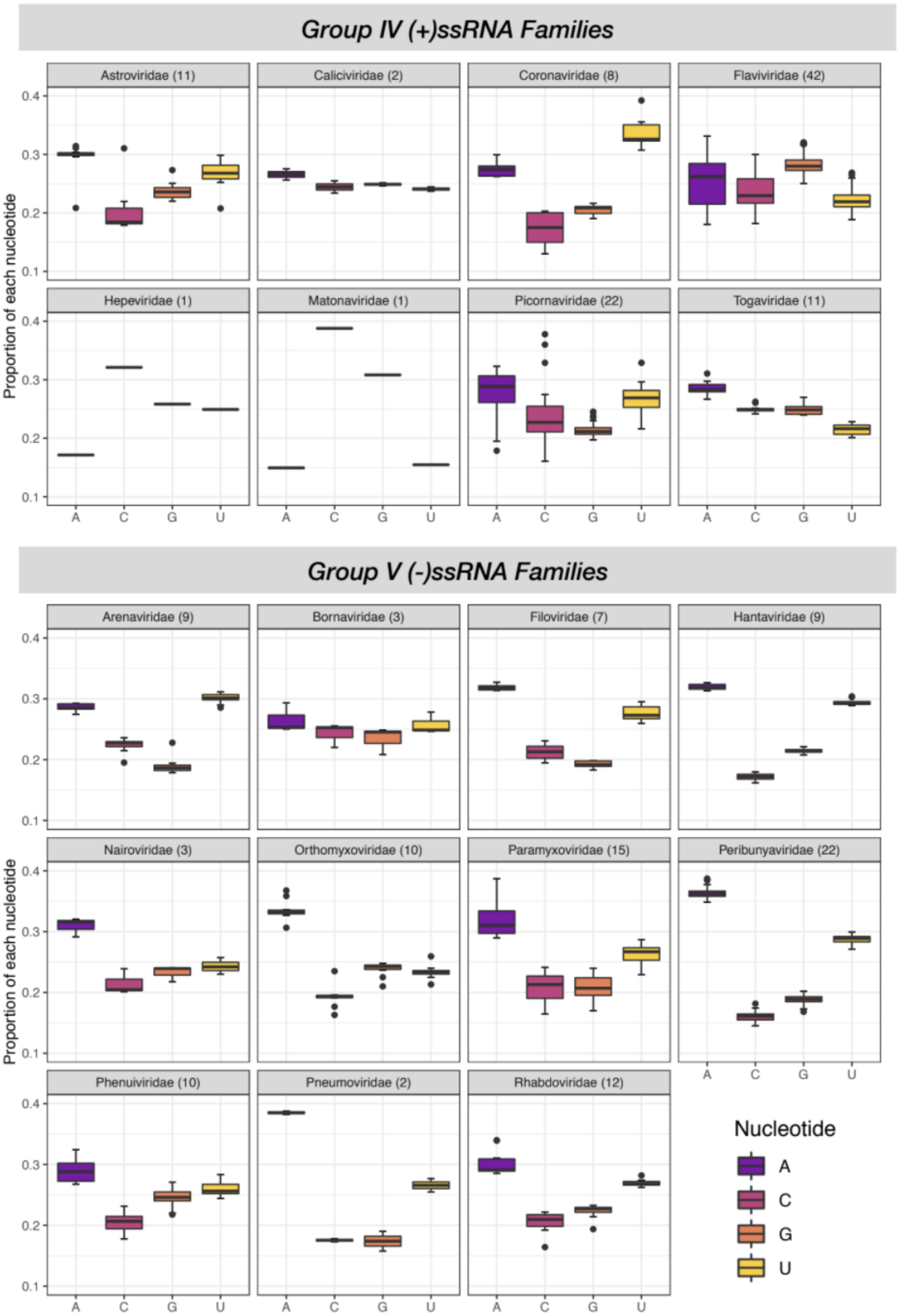

Supplementary Figure S3: Boxplots showing base composition of the (+) sense genome in different viral families. The number of genomes for each family is given in parentheses. Box plots are defined as follows: center line, median; box limits, upper and lower quartiles; whiskers, 1.5x interquartile range; points, outliers.

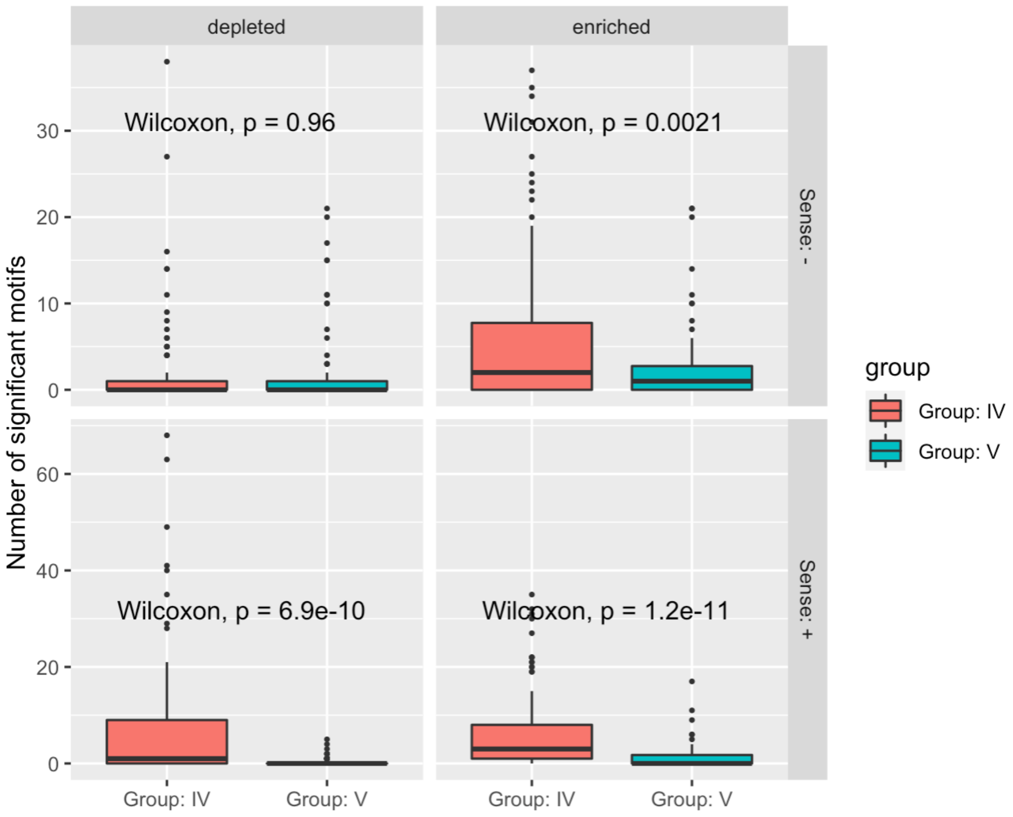

Supplementary Figure S4: Box plots showing the number of motifs in our dataset that were enriched or depleted per genome in Group IV or Group V viruses, on the + or - sense sequences. Box plots are defined as follows: center line, median; box limits, upper and lower quartiles; whiskers, 1.5x interquartile range; points, outliers.

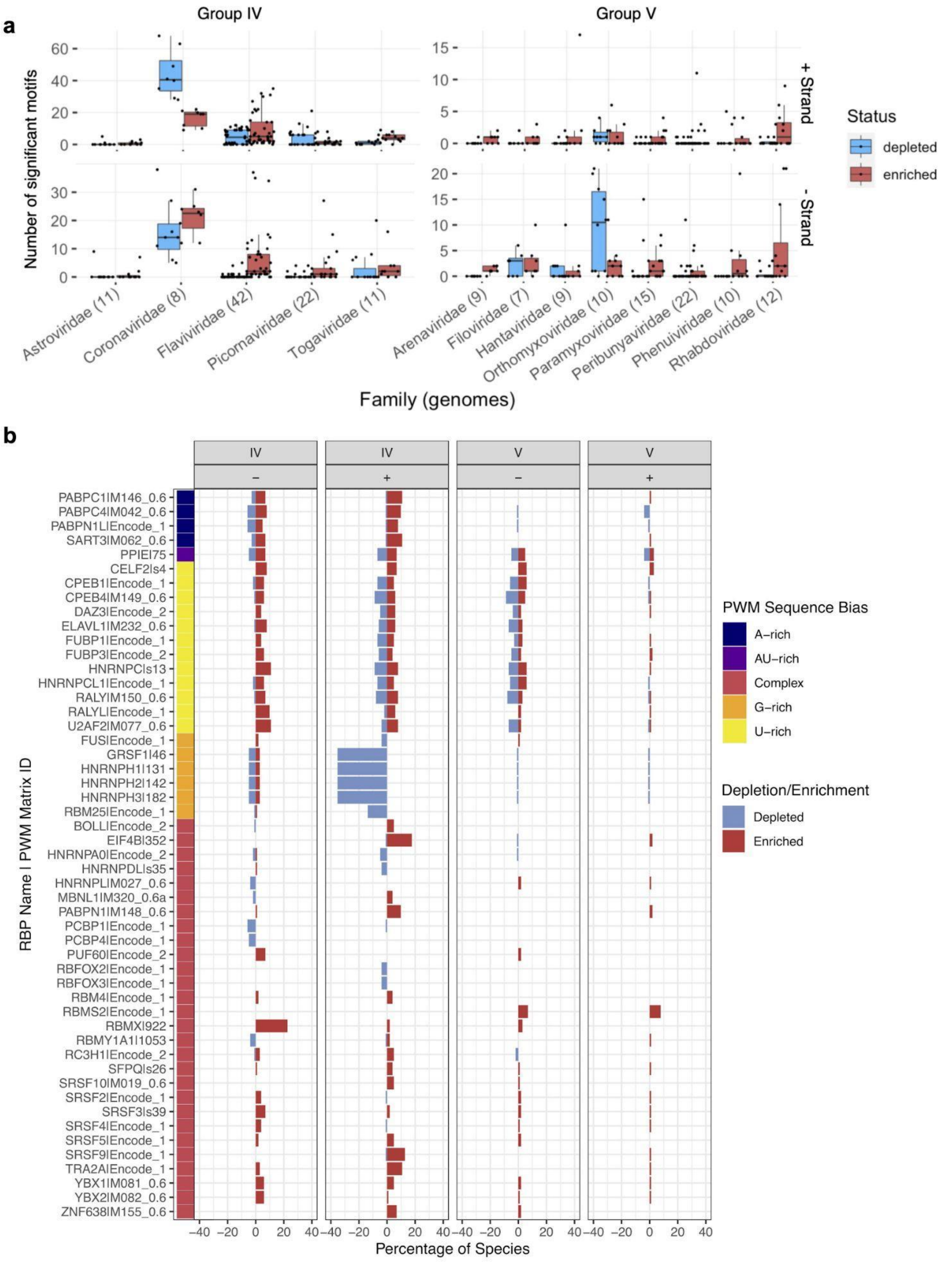

Supplementary Figure S5: SMEAGOL uncovers RBPs whose binding motifs are enriched or depleted in ssRNA virus genomes. a | Number of motifs significantly enriched or depleted in each viral family belonging to Groups IV and V. The number of genomes per family is given in parentheses. Families with more than 5 genomes in our dataset were included. Box plots are defined as follows: center line, median; box limits, upper and lower quartiles; whiskers, 1.5x interquartile range. Individual data points are also shown. b | Percentage of

viral genomes with significant (two-sided binomial test, FDR-adjusted p-values < 0.05) enrichment (in red) and depletion (in blue) per PWM, separated by virus class (IV or V) and viral genome strand. For readability, shown are only representative PWMs that had more than three significant enrichment / depletion events in at least one of the populations (group IV or V in strand + or -). PWM sequence biases (see Methods) are presented on the left side of the plot. While some PWMs have a more complex sequence (light red), others are rich in single nucleotides (A-rich in navy blue, AU-rich in purple, U-rich in yellow, and G-rich in orange). A comprehensive figure that contains all representative PWMs is provided as Supplementary Fig. S6.

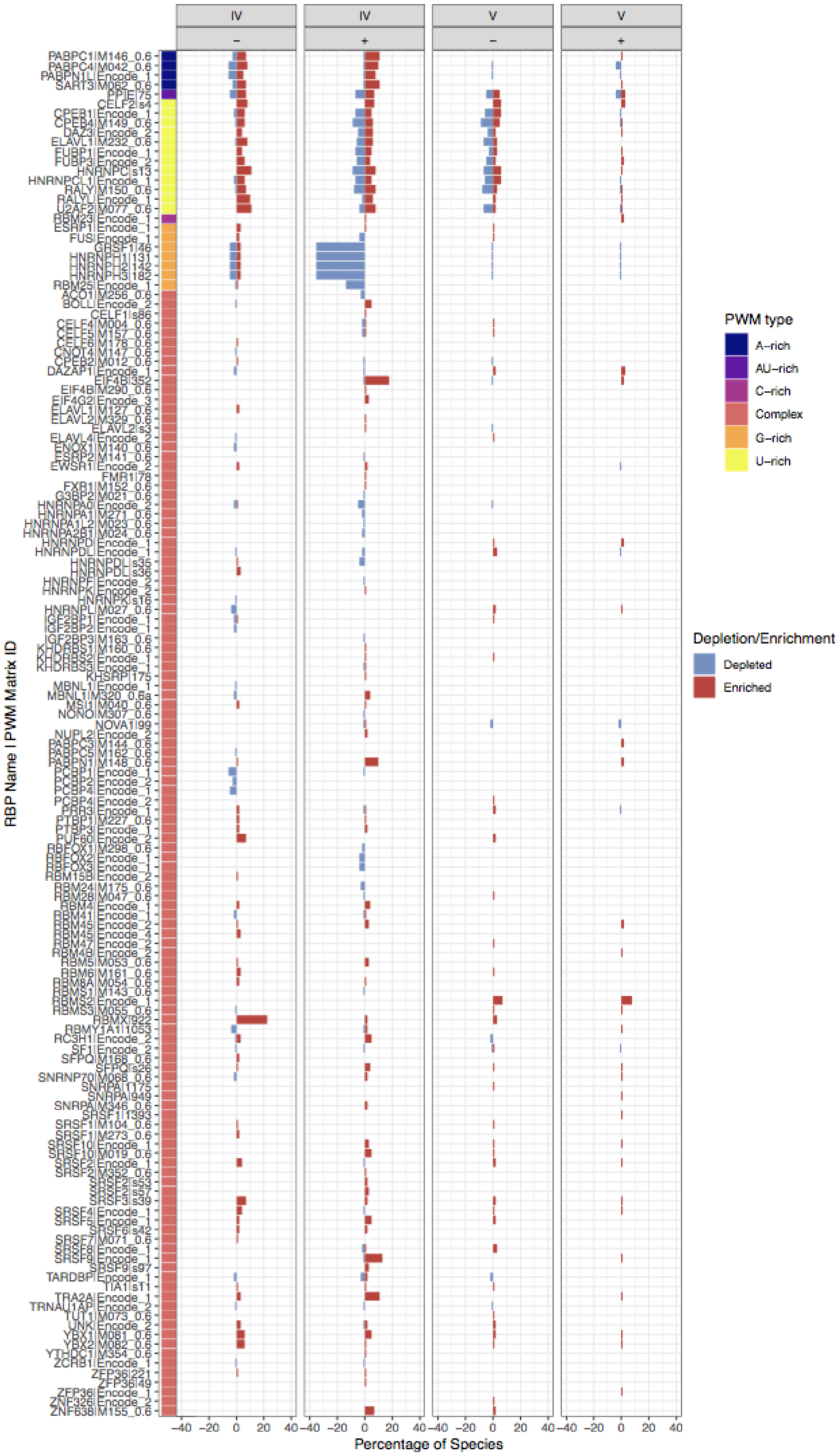

Supplementary Figure S6: Percentage of viral genomes with significant (two-sided binomial test, FDR-adjusted p-values < 0.05) enrichment (in red) and depletion (in blue) per PWM, separated by virus class (IV or V) and viral genome strand, for all representative PWMs. PWM sequence biases (see Methods) are presented on the left side of the plot: while some PWMs have a more complex sequence (light red), others are rich in single nucleotides (A-rich in navy blue, AU-rich in purple, U-rich in yellow, and G-rich in orange).

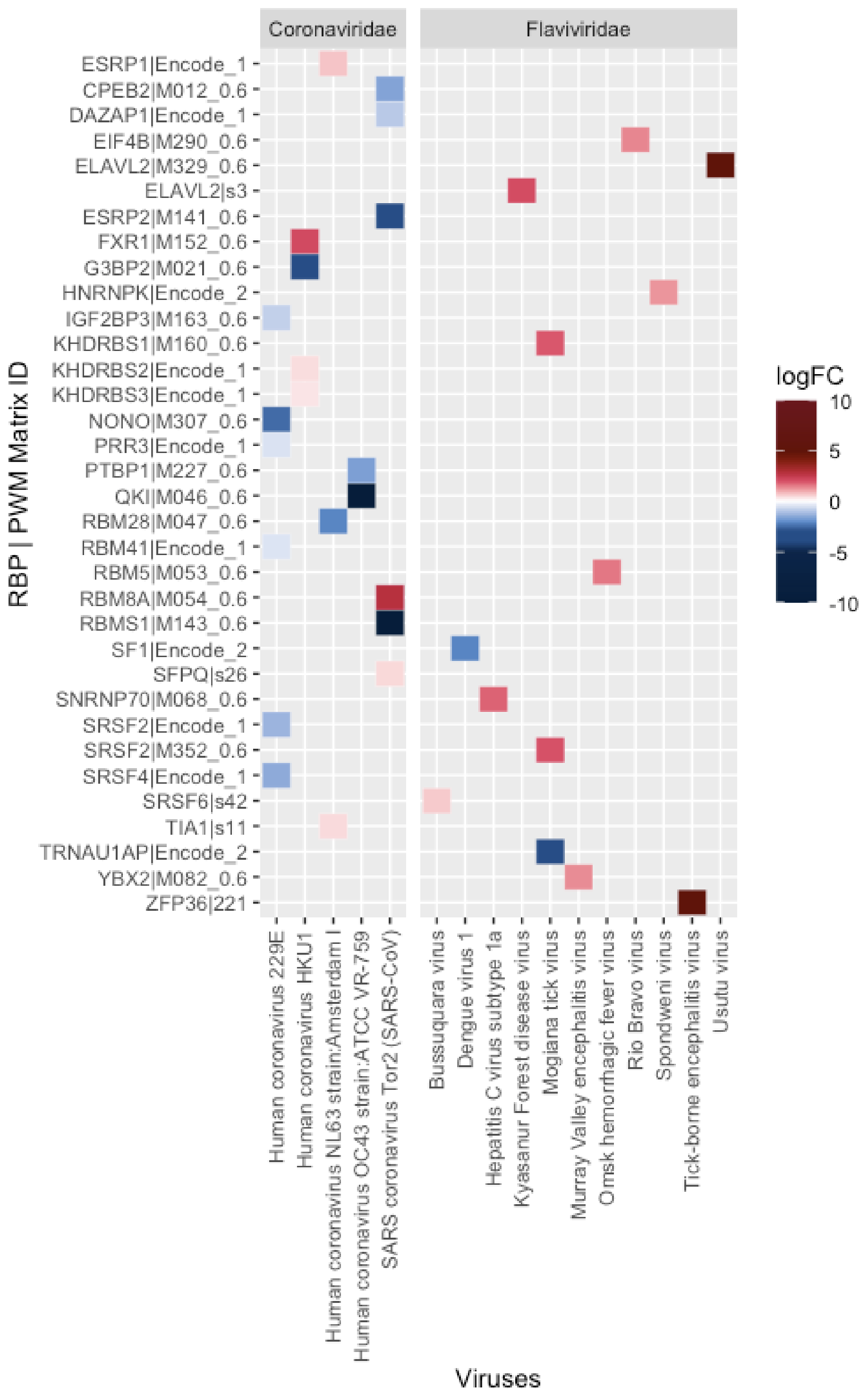

Supplementary Figure S7: Heatmap for species-specific enrichment / depletion log2 fold changes (LogFC) within the *Coronaviridae* and *Flaviviridae* families.

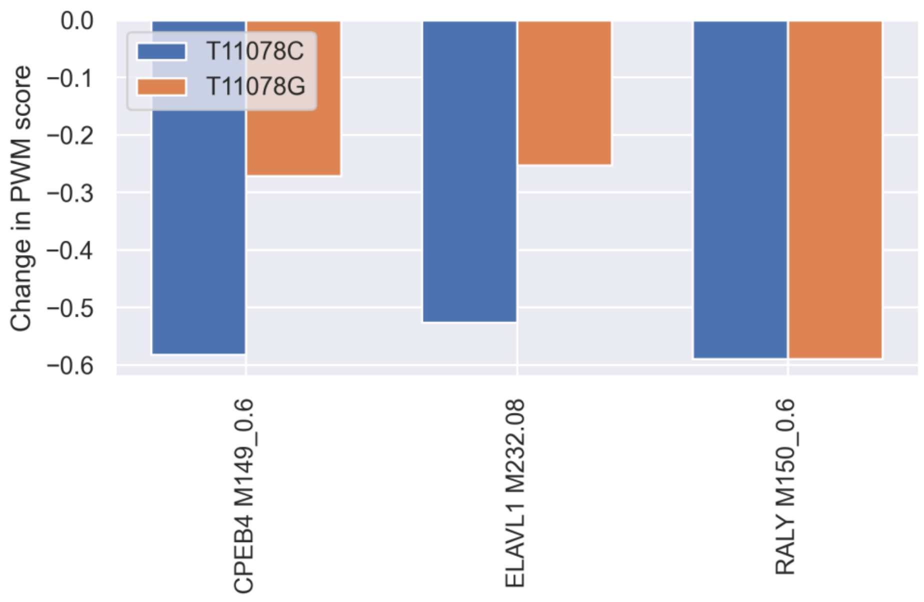

Supplementary Figure S8: Predicted effect of T>C and T>G mutations at position 11078 of the SARS-CoV-2 genome, on binding of three RBPs (CPEB4, ELAVL1 and RALY).

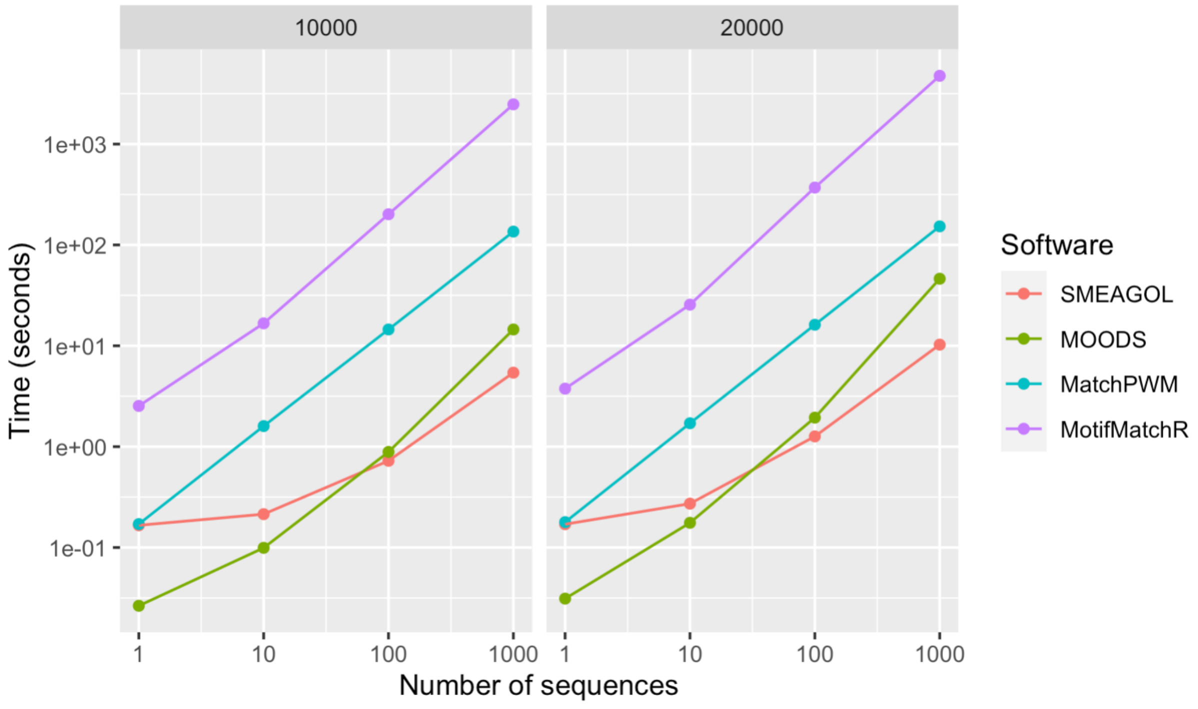

Supplementary Figure S9: Time in seconds to scan groups of DNA sequences with 50 PWMs, using the `smeagol.scan.scan_sequences` function of SMEAGOL, or other PWM scanning softwares. The x-axis shows the number of sequences scanned while the header shows the sequence length in number of bases. Benchmarks were performed on a compute node with 28 CPUs and 252 GB of memory. SMEAGOL v0.0.1, MOODS 1.9.4.1, Biostrings (MatchPWM) 2.54.0, and motifmatchr 1.8.0 were used for this comparison.

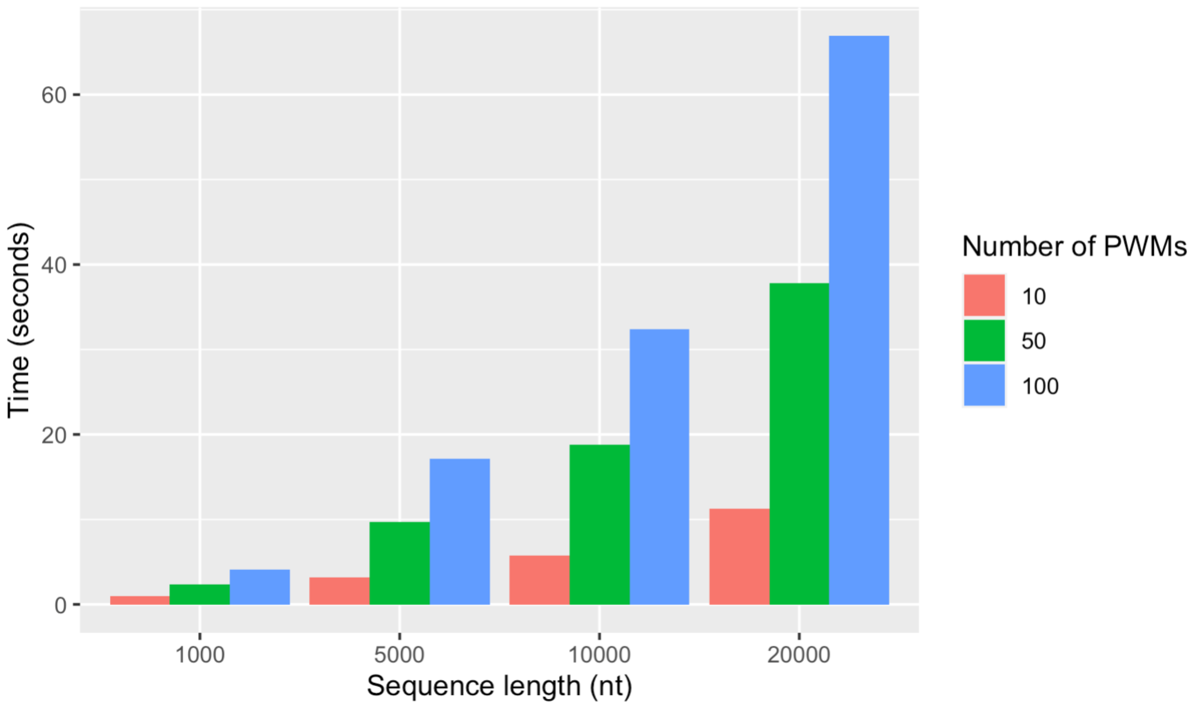

Supplementary Figure S10: Time in seconds to perform complete enrichment analysis using the `smeagol.enrich.enrich_in_genome` function of SMEAGOL for given numbers of PWMs, on a single sequence of length 1000, 5000, 10000 or 20000 bases. The enrichment analysis includes motif scanning, constructing a dinucleotide-shuffled background for the sequence, statistical testing for enrichment or depletion of motifs relative to the background, and multiple hypothesis testing correction of p-values. Benchmarks were performed on a node with 28 CPUs and 252 GB of memory.
